# Lenvatinib Is Highly Effective in Patients with Hepatocellular Carcinoma Related to Both Metabolic Dysfunction-Associated Steatohepatitis and Alcoholic Etiology: A Propensity Score Analysis

**DOI:** 10.3390/cancers17111808

**Published:** 2025-05-28

**Authors:** Rodolfo Sacco, Edoardo G. Giannini, Raffaella Tortora, Giovan Giuseppe Di Costanzo, Andrea Mega, Luca Marzi, Giulia Pieri, Andrea Pasta, Bruno Daniele, Piera Federico, Giuseppe Cabibbo, Maurizio Russello, Caterina Cocuzza, Luca Giacomelli, Marianna Silletta, Paolo Gallo, Umberto Vespasiani Gentilucci, Andrea Casadei-Gardini, Ernesto Claar, Adriano Pellicelli, Massimo Bellini, Filomena Morisco, Concetta Tatali, Valeria Pace Palitti, Antonio Izzi, Marco Di Stefano, Luca Rinaldi, Antonio Facciorusso

**Affiliations:** 1 Gastroenterology and Digestive Endoscopy Unit, Department of Surgical and Medical Sciences, University of Foggia, Viale Pinto 1, 71122 Foggia, Italy; concetta.tatali@gmail.com; 2 Gastroenterology Unit, Department of Internal Medicine, University of Genova, Istituto di Ricovero e Cura a Carattere Scientifico (IRCCS) Ospedale Policlinico San Martino, 16126 Genova, Italy; egiannini@unige.it (E.G.G.); giulia.pieri@hsanmartino.it (G.P.); andrea.pasta@edu.unige.it (A.P.); 3 Liver Unit, Department of Transplantation, Cardarelli Hospital, 80131 Naples, Italy; raffellatortora@live.com (R.T.); giovangiuseppe.dicostanzo@aocardarelli.it (G.G.D.C.); 4 Gastroenterology Unit, Bolzano Regional Hospital, 39100 Bolzano, Italy; andrea.mega@sabes.it (A.M.); luca.marzi@sabes.it (L.M.); 5 Medical Oncology Unit, Ospedale del Mare, 80147 Naples, Italy; b.daniele@libero.it (B.D.); pierafederico@yahoo.it (P.F.); 6 Section of Gastroenterology & Hepatology, Department of Health Promotion, Mother and Child Care, Internal Medicine and Medical Specialties, PROMISE, University of Palermo, 90127 Palermo, Italy; giuseppe.cabibbo78@gmail.com; 7 Liver Unit, ARNAS Garibaldi-Nesima, 95122 Catania, Italy; mrussello@arnasgaribaldi.it (M.R.); ccocuzza@arnasgaribaldi.it (C.C.); 8 Polistudium SRL, 20121 Milan, Italy; luca.giacomelli@polistudium.it; 9 Division of Medical Oncology, Fondazione Policlinico Universitario Campus Bio-Medico, 00128 Rome, Italy; m.silletta@policlinicocampus.it; 10 Clinical Medicine and Hepatology Unit, Fondazione Policlinico Universitario Campus Bio-Medico, 00128 Rome, Italy; paolo.gallo@policlinicocampus.it (P.G.); u.vespasianigentilucci@piliclinicocampus.it (U.V.G.); 11 Department of Oncology, Vita-Salute San Raffaele University, IRCCS San Raffaele Scientific Institute Hospital, 20132 Milan, Italy; casadeigardini@gmail.com; 12 Department of Medicine, Ospedale Evangelico Villa Betania, 80147 Naples, Italy; ernestoclaar@libero.it; 13 Liver Unit, Department of Liver Transplant, A.O. San Camillo Forlanini, 00152 Rome, Italy; adriano.pellicelli@uniroma1.it; 14 Gastroenterology Unit, University of Pisa, 56124 Pisa, Italy; massimo.bellini@unipi.it; 15 Department of Clinical Medicine and Surgery, University of Naples “Federico II”, 80131 Naples, Italy; filomena.morisco@unina.it; 16 Internal Medicine and Hepatology Unit, ASL Pescara, 65124 Pescara, Italy; vpacepalitti@gmail.com; 17 Emergency and Highly Contagious Infectious Diseases, A.O. dei Colli, P.O.D. Cotugno, 80131 Naples, Italy; izziantonio@yahoo.it; 18 Hepatology Unit, Umberto I Hospital, 00161 Siracusa, Italy; marcodistefano1964@gmail.com; 19 Department of Medicine and Health Sciences, University of Molise, 86100 Campobasso, Italy; luca.rinaldi@unimol.it; 20 Gastroenterology Unit, Department of Experimental Medicine, Università del Salento, 73100 Lecce, Italy; antonio.facciorusso@virgilio.it

**Keywords:** HCC, survival, progression, liver cancer, propensity matching

## Abstract

**Background and aims:** Metabolic dysfunction-associated steatotic liver disease (MASLD)-related hepatocellular carcinoma (HCC) may have distinct biological characteristics influencing systemic treatment response. However, the prognostic impact of MASLD vs. alcohol-related HCC in patients receiving lenvatinib remains unclear. This study aimed to assess lenvatinib’s effectiveness and safety in these populations. **Methods:** A multicenter cohort of 378 HCC patients treated with lenvatinib (2019–2024) was analyzed. Propensity score matching was performed based on age, sex, tumoral stage, alpha-fetoprotein levels and Child–Pugh class. Survival was estimated using Kaplan–Meier analysis and compared with the log-rank test. Results were expressed as HR and 95% CI. **Results:** After matching, 115 patients per group were compared. Median OS was 21 months (95% CI: 20–23) in the group with metabolic dysfunction-associated steatohepatitis (MASH) and 19 months (95% CI: 18–21) in the group with alcohol etiology (*p* = 0.18). In multivariate analysis, only Child–Pugh class (HR 2.67, 95% CI: 1.84–5.41) and tumor stage (HR 2.18, 95% CI: 1.57–6.93) resulted as significant predictors of OS. Median PFS was 9 months (95% CI: 8–9) in patients with MASH and 9 months (95% CI: 7–10) in patients with alcohol etiology (*p* = 0.33). Only the Child–Pugh class was a significant predictor of PFS in univariate analysis (HR 1.56, 95% CI: 1.15–3.41; *p* = 0.03). No difference in terms of adverse event rate was observed between the two groups. **Conclusions:** Lenvatinib is effective in patients with both MASH- and alcohol-related HCC, with no difference in oncological outcomes between the two groups.

## Simple Summary

Liver cancer is a common and serious condition often linked to liver damage caused by alcohol use or a metabolic condition known as MASH, which is related to obesity and diabetes. It is important to understand whether treatments work equally well for different causes of liver disease. In this study, we examined how patients with liver cancer caused by alcohol or MASH responded to lenvatinib, a drug used to treat advanced liver cancer. We analyzed data from 230 patients from multiple hospitals and compared outcomes between the two groups. Our findings showed that lenvatinib was equally effective and safe in both groups, with similar survival times and side effect profiles. These results suggest that the underlying cause of liver disease does not affect how well patients respond to lenvatinib. This information can help doctors make better treatment decisions for people with liver cancer.

## 1. Introduction

Hepatocellular carcinoma (HCC) is the fifth most common cancer worldwide and the most frequent cause of mortality in patients with cirrhosis [1].

Despite, in recent years, an increased use of surveillance leading to more frequent diagnoses of patients with HCC at earlier stages, and who are thus amenable to therapies with curative intention, a vast proportion of patients—approximately 40%—are still diagnosed with multinodular tumors, with extra-hepatic spread or macrovascular invasion, and are therefore amenable to systemic therapy alone [2].

In these patients, the oral multikinase inhibitor sorafenib represented for a long time the only available first-line systemic treatment, and it is still frequently used in this setting [3,4]. However, more recently, several alternative options have been developed, such as lenvatinib, or immunotherapy with a combination of atezolizumab and bevacizumab or tremelimumab and durvalumab, which improved the clinical management of patients with advanced HCC [5,6,7]. Specifically, lenvatinib showed favorable results both in randomized-controlled trials (RCTs) and in real-life series [5,8,9,10]. Moreover, a recent meta-analysis confirmed similar overall survival (OS) and more favorable progression-free survival (PFS) compared to sorafenib in first-line therapy [11]. Real-world studies indicate that, as far as OS is concerned, lenvatinib is as effective as atezolizumab–bevacizumab, and that in Child–Pugh B patients, who represent a difficult-to-treat population, OS and PFS were similar between treatments, reinforcing lenvatinib as a strong alternative to immunotherapy in this setting [12,13,14].

Data from retrospective studies should be interpreted with caution due to several potential confounders that should be considered in the analysis; some previous studies identified different potential clinical prognostic factors to predict the response to lenvatinib, such as body mass index (BMI) [15], cachexia [16] and non-viral etiology [17].

Noteworthily, etiology related to non-alcoholic steatohepatitis (NASH), with most cases now redefined as metabolic dysfunction-associated steatohepatitis (MASH), was found to worsen prognosis in patients treated with immune checkpoint inhibitors, probably due to a different hepatic microenvironment with consequent different responsiveness to certain treatments [18,19]. Conversely, lenvatinib has demonstrated favorable outcomes in patients with MASH-related HCC, suggesting potential differences in tumor biology and treatment response depending on etiology [20]. However, the prognostic impact of metabolic dysfunction-associated steatotic liver disease compared to alcohol-related HCC in patients receiving lenvatinib remains unclear.

To properly assess this important clinical issue and to better identify the prognostic role of the etiology of the underlying liver disease, we conducted a propensity score-matching analysis in a large, multicenter series of patients with HCC treated with lenvatinib as first-line therapy, aiming to compare the oncological outcomes of different etiologies, namely MASH and alcoholic etiology, in this setting.

## 2. Methods

### 2.1. Patients

A multicenter, prospectively compiled dataset was analyzed, comprising patients with intermediate to advanced HCC who were ineligible for surgical resection or locoregional treatment and received first-line lenvatinib therapy between May 2019 and June 2024.

In all cases, HCC was diagnosed based on histology and/or imaging in accordance with international guidelines [2,21].

Eligible patients met the following criteria: an ECOG performance status (PS) score of 2 or less; a Child–Pugh classification not exceeding B7; a platelet count of at least 50 × 10^9^/L; hemoglobin levels ≥8.5 g/dL; a prothrombin time international normalized ratio (INR) ≤ 2; and preserved renal function, defined as a serum creatinine concentration no greater than 1.5 times the upper limit of normal. All patients provided written informed consent before their enrolment in the study. This study was approved by the ethics committee of the leading center.

### 2.2. Treatment

Oral lenvatinib was administered once daily, with a dosage of 12 mg for patients weighing ≥60 kg and 8 mg for those under 60 kg at baseline.

Radiological assessment of tumor response was conducted via computed tomography (CT) or magnetic resonance imaging (MRI) every 8 weeks, or earlier if clinically indicated, following the modified RECIST (mRECIST) criteria [22].

Treatment discontinuation occurred upon evidence of disease progression or in cases of intolerable toxicity. Dose adjustments were made on a case-by-case basis in response to adverse events (AEs).

### 2.3. Outcomes

The main endpoint was OS, defined as the time from initiation of therapy to either death or last follow-up (censoring). Secondary endpoints included PFS, measured from treatment initiation to radiologically confirmed disease progression; objective response (OR), encompassing both complete and partial responses; and the incidence of adverse events (AEs).

The intensity of AEs was classified in accordance with version 4.0 of the Common Terminology Criteria for Adverse Events (CTCAE) [23].

### 2.4. Statistical Analysis

Descriptive statistics for categorical data were expressed as absolute counts and proportions, whereas continuous variables were summarized using the median and interquartile range (IQR). Survival analyses were conducted using the Kaplan–Meier method, with outcomes presented as medians alongside their 95% confidence intervals (CIs). Group comparisons for time-to-event endpoints were performed using the log-rank test. The inferential analysis for time-to-event data was conducted using the Cox univariate and multivariate regression model and reported in terms of hazard ratios (HRs) and 95% CI. Statistically significant variables from the univariate Cox analysis were consistently included in the multivariate models.

To overcome biases related to the different distribution of covariates among patients with MASH or alcohol-associated HCC, a 1-to-1 match was created using propensity score analysis. The propensity score represents the probability of each patient being assigned to a particular condition or treatment in a study given a set of known covariates [24].

A multivariate logistic regression model was built to predict the probability of each patient falling into the two groups based on several demographic and collection-related covariates, including age, sex, tumoral stage, alpha-fetoprotein (AFP) levels and Child–Pugh score.

To match the two groups, the nearest neighbor method was used, which selects as a match a control whose propensity score is closest to that of the case (“nearest neighbor matching” approach), with the further restriction that the absolute difference in the propensity scores of matched subjects must be below some prespecified threshold (the caliper distance) [25]. As described elsewhere, a caliper of width equal to 0.2 of the standard deviation of the logit of the propensity score was used [26].

The analysis was performed using the matchit package of R Statistical Software 4.2.3 (Foundation for Statistical Computing, Vienna, Austria), and significance was established at the 0.05 level (two-sided).

## 3. Results

### 3.1. Baseline Characteristics of Patients

**Figure 1:**
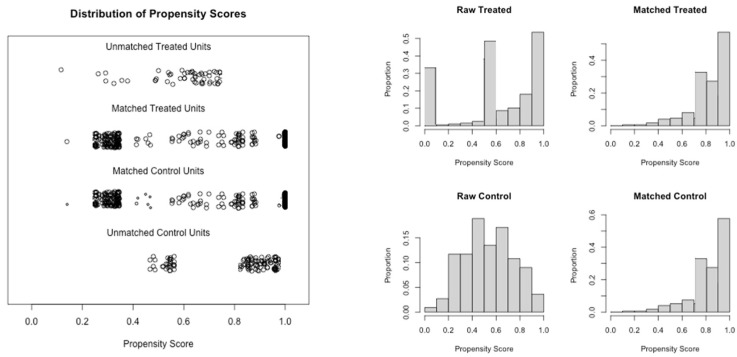
Details of the propensity score matching model. (**Left Panel**) Jitter plot. (**Right Panel**) Histogram.

Out of 378 patients initially assessed for eligibility, of which 227 had MASH and 151 had alcohol etiology, after 1-to-1 propensity score matching, 230 patients were selected for comparison: 115 with MASH and 115 with alcohol etiology. Details of the propensity score matching are shown in the left and right panels of Figure 1. The characteristics of the 230 propensity score-matched patients are reported in Table 1.

The median age was 73 years in both cohorts, with an interquartile range of 58–80 years in the MASH group and 55–81 years in the alcohol-related group (*p* = 0.87). The majority of patients were male in both groups (74.7%), with no significant difference observed (*p* = 1.0). An ECOG performance status of 0 was recorded in 81.7% of patients with MASH and 80% of those with alcohol-related HCC (*p* = 0.84). Most participants in both groups were classified as Child–Pugh class A (89.5%, *p* = 0.76), and 60% were categorized as Barcelona Clinic Liver Cancer (BCLC) stage C (*p* =1.0). The median alpha-fetoprotein (AFP) levels were 257.3 IU/mL (IQR: 135.1–578) in the MASH group and 253.4 IU/mL (IQR: 135–618.3) in the alcohol group (*p* = 0.75). Additionally, 60% of patients in both groups had undergone prior radical or locoregional treatments (*p* = 1.0).

Among patients with alcohol-related etiology, 24 individuals (20.8%) had a concurrent viral hepatitis infection.

### 3.2. Overall Survival

**Figure 2:**
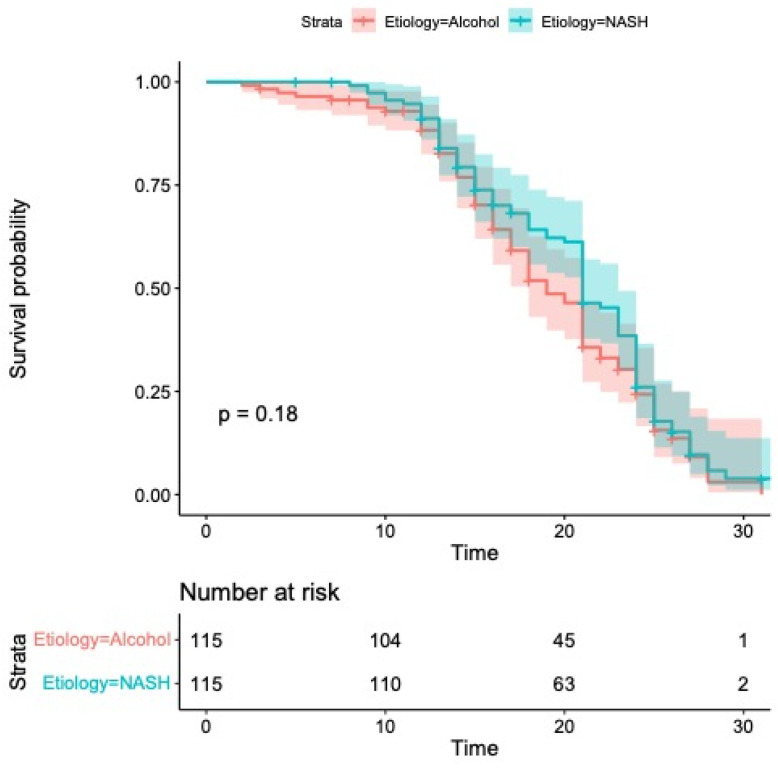
Kaplan–Meier curves comparing overall survival. Median overall survival was 21 months (95% CI: 20–23) in the group with MASH and 19 months (95% CI: 18–21) in the group with alcohol etiology (*p* = 0.18).

Median OS was 21 months (95% CI: 20–23) in the group with MASH and 19 months (95% CI: 18–21) in the group with alcohol etiology (*p* = 0.18; Figure 2).

According to the univariate analysis presented in Table 2, overall survival (OS) was significantly influenced by Child–Pugh classification (HR 2.43, 95% CI: 1.92–6.18; *p* < 0.001), alpha-fetoprotein (AFP) levels (HR 2.17, 95% CI: 1.08–4.10; *p* = 0.01), BCLC staging (HR 2.27, 95% CI: 1.43–6.10; *p* = 0.001) and ECOG performance status (HR 1.77, 95% CI: 1.02–3.10; *p* = 0.04). Multivariate modeling confirmed Child–Pugh class (HR 2.67, 95% CI: 1.87–5.41; *p* < 0.001) and BCLC stage (HR 2.18, 95% CI: 1.57–6.93; *p* = 0.001) as independent prognostic factors for OS. Importantly, no statistically significant difference in OS was detected between patients with MASH and those with alcohol-related HCC (*p* = 0.20).

### 3.3. Progression-Free Survival

**Figure 3:**
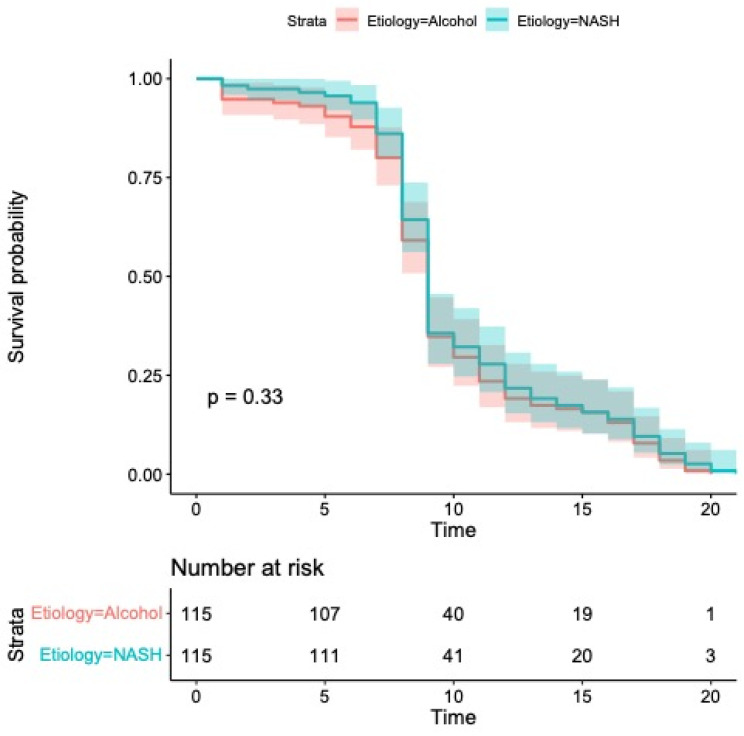
Kaplan–Meier curves comparing progression-free survival. Median progression-free survival was 9 months (95% CI: 8–9) in patients with MASH and 9 months (95% CI: 7–10) in patients with alcohol etiology (*p* = 0.33).

As illustrated in Figure 3, the median progression-free survival (PFS) was 9 months in both groups—ranging from 8 to 9 months in the MASH cohort and from 7 to 10 months among patients with alcohol-related HCC (*p* = 0.33).

Univariate analysis identified the Child–Pugh classification as the sole significant predictor of PFS (HR 1.56, 95% CI: 1.15–3.41; *p* = 0.03; Table 3), which precluded the need for multivariate modeling. Consistent with overall survival outcomes, PFS did not differ significantly by underlying disease etiology (HR 0.88, 95% CI: 0.68–1.15; *p* = 0.36; Table 3).

### 3.4. Other Secondary Outcomes

Tumor response rates were comparable between the two cohorts, with objective response (OR) observed in 39.8% of patients in the MASH group and 40.3% in those with alcohol-related etiology (*p* = 0.72).

The incidence of adverse events (AEs) of any grade was also similar, occurring in 104 patients (90.5%) with MASH and 105 patients (91.4%) with alcohol-associated disease (*p* = 0.54). The most frequently reported AEs included hand–foot skin reaction (20.3%), hypertension (11.5%) and diarrhea (10%). Grade 3 or 4 AEs were recorded in 13.5% of MASH patients and 15% of alcohol-related cases, with no significant difference between groups (*p* = 0.69).

## 4. Discussion

HCC represents a major health issue and the most frequent cause of tumor-related mortality in patients with cirrhosis [1]. Lenvatinib is able to increase the survival of patients with advanced HCC, although which subsets of patients would benefit most from this treatment is still unclear. Given the widespread use of lenvatinib in patients with HCC, clarifying its efficacy across different etiologies may be relevant to individualize treatment selection.

A recent multicenter retrospective series found both OS and PFS statistically improved in patients with NASH treated with lenvatinib [19], thus pointing out a potential negative prognostic role of other etiologies, such as viral-related cirrhosis, in this setting.

Recent terminology changed the concept of NASH to MASH to avoid the use of exclusionary confounder terms and potentially stigmatizing language [27]. The progressive rise of obesity prevalence in the West in recent years has, therefore, led to a dramatic increase in MAFLD/MASH incidence, with a consequent growth of MASH-driven HCC, and it is expected that this incidence will continue to rise internationally in the near future. Hence, the identification of MASH as a potential prognostic factor in HCC patients represents a major issue in this field.

This notwithstanding, both registration trials and several real-world studies analyzed patients with non-viral etiology without a clear distinction between patients with metabolic dysfunction-associated steatotic liver disease/MASH and patients with alcohol etiology. Therefore, in this regard, we deemed it of interest to assess, in a large cohort of Western patients treated with lenvatinib, the potential presence of different outcomes in patients with non-viral etiology by adequately stratifying them into MASH- and alcohol-related etiology.

Thus, to better understand the prognostic role of the etiology of the underlying liver disease in this setting, we performed a propensity score matching analysis comparing two groups of patients with HCC treated with lenvatinib, namely MASH patients and subjects with alcohol etiology. Median OS was about 20 months in our cohort (21 months in the group with MASH and 19 months in the group with alcohol etiology), and therefore in line with the available literature [12,14] and with no difference between the two study groups. Among the several tested prognostic factors, only the Child–Pugh class and BCLC stage were significant predictors of OS in multivariate analysis. Likewise, only the Child–Pugh class was a significant predictor of PFS in univariate analysis, with no difference observed between MASH and alcohol-related HCC (*p* = 0.36).

The underlying pathways leading to the transformation of MASH in HCC, although still not completely understood, are likely to be driven mainly by the inflammatory status of the microenvironment, with an increased oxidative stress leading to aberrant liver regeneration [28]. A similar pathway was also observed in other metabolic-associated conditions [24], and might explain the worse response of immunotherapy in MASH-related HCC patients, unlike other treatments, such as lenvatinib [19,20].

Our series represents the first study directly comparing MASH and alcohol-related HCC patients, and it shows that lenvatinib is effective in HCC patients with both etiologies. Indeed, lenvatinib acts by inhibiting multiple receptor tyrosine kinases, including VEGFR1–3, FGFR1–4, PDGFRα, RET and KIT, thereby impairing angiogenesis and tumor proliferation. Its broad mechanism of action, less dependent on immune modulation, may explain its consistent efficacy across different HCC etiologies, including MASH-related disease, where immune checkpoint inhibitors, according to some studies, seem to have reduced effectiveness [16,18,19]. This supports lenvatinib’s role as a robust therapeutic option irrespective of underlying liver disease.

Furthermore, no difference in terms of all the oncological outcomes, including also tumor response and AE rate, was observed between the two study groups and toxicity data were in keeping with the current literature [8,9,10,11,12].

Our study presents some limitations. First, we performed a retrospective observational study on a large population, and we could not exclude a potential selection bias. However, a robust propensity score matching model was built, accounting for all the main potential confounders in this analysis. Second, the multicenter nature of the analysis did not allow for the centralization of imaging, and the criteria for treatment and re-evaluation were consistent with the clinical practice of each center and in accordance with national guidelines. Third, a classification bias due to under-declaration of alcohol and tobacco consumption or drug abuse could represent a limitation to our study. Last, emerging biomarkers, such as fibroblast growth factor 21 (FGF21), may offer valuable insight into the metabolic drivers of hepatocarcinogenesis and could serve to further refine patient stratification in future studies [28]. However, FGF21 measurements were not available in our retrospective cohort, and prospective studies are needed to explore its prognostic and predictive role in patients treated with systemic therapies.

## 5. Conclusions

In conclusion, our results show the favorable effects of lenvatinib in first-line therapy in patients with advanced HCC with either MASH or alcohol etiology of liver disease. As patients with these underlying conditions are expected to represent the largest share of patients with HCC in the future [27], we feel that our results may be of direct clinical interest. Beyond lenvatinib, the therapeutic landscape for advanced HCC includes other tyrosine kinase inhibitors such as cabozantinib and regorafenib, used predominantly in second-line settings after sorafenib failure. Additionally, the combination of immune checkpoint inhibitors with anti-angiogenic agents, particularly atezolizumab plus bevacizumab, or the combination of two immune checkpoint inhibitors, such as tremelimumab and durvalumab, has emerged as a standard first-line treatment option. Nonetheless, lenvatinib remains an important first-line alternative, particularly in patients with contraindications to immunotherapy or those with impaired liver function, highlighting the clinical relevance of our findings across diverse patient populations.

## Figures and Tables

**Table 1:** Baseline characteristics of the patients after propensity score matching.

Variable	MASH (*n* = 115)	Alcohol (*n* = 115)	*p*-Value
Age (years)	73 (58–80)	73 (55–81)	0.87
Sex (Male)	86 (74.7%)	86 (74.7%)	1.0
ECOG PS 0	94 (81.7%)	92 (80%)	0.84
Child–Pugh:			0.76
Class A	103 (89.5%)	103 (89.5%)	
Class B	12 (10.5%)	12 (10.5%)	
BCLC:			1.0
Stage B	46 (40%)	46 (40%)	
Stage C	69 (60%)	69 (60%)	
AFP (UI/mL)	257.3 (135.1–578)	253.4 (135–618.3)	0.75
HBV/HCV infection	–	24 (20.8%)	–
Previous radical or locoregional therapy	69 (60%)	69 (60%)	1.0

Values are expressed as median (interquartile range) or absolute numbers (%) when appropriate. AFP: alpha-fetoprotein; BCLC: Barcelona Cancer of the Liver Clinic; ECOG: Eastern Cooperative Oncology Group; MASH: metabolic dysfunction-associated steatohepatitis; PS: performance status.

**Table 2:** Cox univariate/multivariate regression for overall survival.

Variables	Univariate Analysis		Multivariate Analysis	
	HR (95% CI)	*p*-Value	HR (95% CI)	*p*-Value
Age (reference ≤ 65 years)	0.82 (0.58–1.33)	0.34		
Sex (reference female)	1.18 (0.52–3.4)	0.34		
Etiology (reference alcohol)	0.82 (0.61–1.11)	0.20		
Child–Pugh class (reference A)	2.43 (1.92–6.18)	**<0.001**	2.67 (1.84–5.41)	**<0.001**
AFP (reference ≤ 400 UI/mL)	2.17 (1.08–4.1)	**0.01**	2.15 (0.75–3.7)	0.10
BCLC stage (reference B)	2.27 (1.43–6.1)	**0.001**	2.18 (1.57–6.93)	**0.001**
Diabetes (reference no)	1.13 (0.7–2.7)	0.41		
HBV/HCV infection (reference no)	1.22 (0.56–3.21)	0.18		
ECOG PS (reference 0)	1.77 (1.02–3.1)	**0.04**	2.11 (0.63–3.15)	0.45

Bold *p*-values are significant. AFP: alpha-fetoprotein; BCLC: Barcelona Cancer of the Liver Clinic; ECOG: Eastern Cooperative Oncology Group; HBV: hepatitis B virus; PS: performance status.

**Table 3:** Cox univariate regression for progression-free survival.

Variables	Univariate Analysis	
	HR (95% CI)	*p*-Value
Age (reference ≤ 65 years)	0.94 (0.45–1.18)	0.51
Sex (reference female)	1.18 (0.71–3.45)	0.76
Etiology (reference alcohol)	0.88 (0.68–1.15)	0.36
Child–Pugh class (reference A)	1.56 (1.15–3.41)	**0.03**
AFP (reference ≤ 400 UI/mL)	2.39 (0.43–5.21)	0.21
BCLC stage (reference B)	1.9 (0.93–2.6)	0.07
Diabetes (reference no)	1.15 (0.8–3.2)	0.78
HBV/HCV infection (reference no)	1.28 (0.8–2.24)	0.45
ECOG PS (reference 0)	1.48 (0.75–2.6)	0.35

Bold *p*-values are significant. AFP: alpha-fetoprotein; BCLC: Barcelona Cancer of the Liver Clinic; ECOG: Eastern Cooperative Oncology Group; HBV: hepatitis B virus; PS: performance status.

## Data Availability

All data are available from the corresponding author upon reasonable request.
